# The CREB/KMT5A complex regulates PTP1B to modulate high glucose-induced endothelial inflammatory factor levels in diabetic nephropathy

**DOI:** 10.1038/s41419-021-03629-4

**Published:** 2021-03-29

**Authors:** Ting Huang, Xue Li, Fei Wang, Lihong Lu, Wenting Hou, Minmin Zhu, Changhong Miao

**Affiliations:** 1 Department of Anesthesiology, Fudan University Shanghai Cancer Center, Shanghai Medical College, Fudan University, Shanghai, 200032 China; 2 Department of Oncology, Shanghai Medical College, Fudan University, Shanghai, 200032 China; 3 Department of Anesthesiology, Zhongshan Hospital, Fudan University, Shanghai, 200032 China

**Keywords:** Diabetes complications, Type 2 diabetes

## Abstract

Diabetic nephropathy (DN) is the primary microvascular complication of diabetes mellitus and may result in end-stage renal disease. The overproduction of various inflammatory factors is involved in the pathogenesis of DN. Protein tyrosine phosphatase 1B (PTP1B) modulates the expression of a series of cytokines and nuclear factor kappa B (NF-κB) activity. cAMP response element-binding protein (CREB) and lysine methyltransferase 5A (KMT5A) have been reported to participate in the maintenance of a healthy endothelium. In the present study, we hypothesise that CREB associates with KMT5A to modulate PTP1B expression, thus contributing to high glucose-mediated glomerular endothelial inflammation. Our analyses revealed that plasma inflammatory factor levels, glomerular endothelial p65 phosphorylation and PTP1B expression were increased in DN patients and rats. In vitro, high glucose increased endothelial inflammatory factor levels and p65 phosphorylation by augmenting PTP1B expression in human umbilical vein endothelial cells (HUVECs). Moreover, high glucose decreased CREB and KMT5A expression. CREB overexpression and KMT5A overexpression both inhibited high glucose-induced PTP1B expression, p65 phosphorylation and endothelial inflammatory factor levels. si-CREB- and sh-KMT5A-induced p65 phosphorylation and endothelial inflammatory factor levels were reversed by si-PTP1B. Furthermore, CREB was associated with KMT5A. Mechanistic research indicated that CREB and histone H4 lysine 20 methylation (H4K20me1, a downstream target of KMT5A) occupy the PTP1B promoter region. sh-KMT5A augmented PTP1B promoter activity and activated the positive effect of si-CREB on PTP1B promoter activity. Our in vivo study demonstrated that CREB and KMT5A were downregulated in glomerular endothelial cells of DN patients and rats. In conclusion, CREB associates with KMT5A to promote PTP1B expression in vascular endothelial cells, thus contributing to hyperglycemia-induced inflammatory factor levels in DN patients and rats.

## Introduction

Diabetic nephropathy (DN) is the primary microvascular complication of diabetes mellitus and may result in end-stage renal disease^1,2^. DN is characterised by a gradual elevation of urine albumin, increased blood pressure, and a decreasing glomerular filtration rate^3^. Limited treatment options are available for DN, and the cost of treatment and cardiovascular-related mortality both increase once DN develops to end-stage renal disease^4,5^. Therefore, identifying the underlying mechanisms of DN is vital. Inflammation has been linked to the pathogenesis of DN, and the overexpression of various inflammatory factors plays a crucial role in the renal reaction to inflammation^6^. Moreover, hyperglycaemia augments glomerular endothelial cell inflammatory factor levels, thus causing renal injury^7^.

Serum and kidney levels of interleukin-1β (IL-1β) are increased in DN patients and participate in the progression of DN^8^. Tumour necrosis factor-α (TNFα) is overexpressed in the serum of diabetic patients, which increases glomerular vasoconstriction and the filtration rate^9^. NF-κB activation plays a crucial role in inflammatory factor expression in DN^10,11^. Moreover, protein tyrosine phosphatase 1B (PTP1B) modulates the expression of a series of cytokines and NF-κB activity^12^. As inflammation develops in DN, fibroblast recruitment ultimately results in renal fibrosis^13^.

cAMP response element-binding protein (CREB) is widely expressed and plays a crucial role in many important cellular signalling pathways, including cellular metabolism, cell cycle progression, survival, and responses to extracellular stimuli^14^. Recent studies indicate that CREB is important for the maintenance of a healthy endothelium^15–18^, but the potential participation of CREB in hyperglycemia-induced inflammatory factor expression has not been comprehensively examined.

Lysine methyltransferase 5A (KMT5A) is the only known nucleosome-specific methyltransferase that modulates histone H4 lysine 20 through methylation (H4K20me1)^19^. We previously demonstrated that KMT5A suppression contributes to high glucose-induced vascular endothelial injury in human umbilical vein endothelial cells (HUVECs)^20–24^. However, the role of KMT5A in high glucose-induced inflammatory factor expression has not been established. In the present study, we hypothesise that CREB cooperates with KMT5A to modulate PTP1B expression and inflammatory factor levels, thus playing a vital role in the occurrence and progression of DN.

## Results

### Plasma inflammatory factor levels and glomerular PTP1B expression are increased in DN patients and rats

**Fig. 1:**
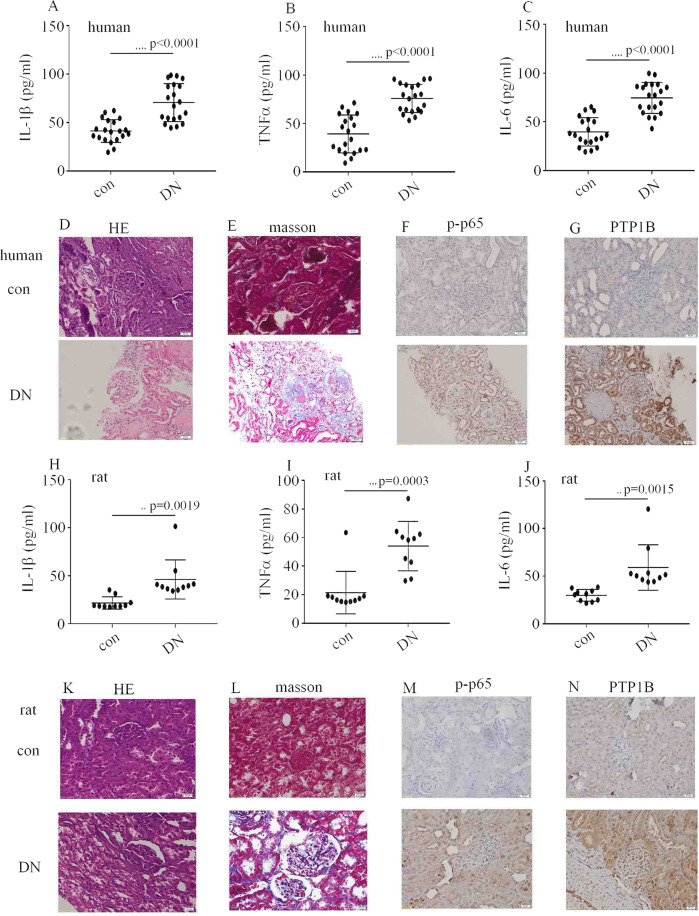
Augmentation of plasma inflammatory factor levels and increased glomerular PTP1B expression in DN patients and rats. **A**–**C** ELISA indicated that IL-1β, TNFα and IL-6 were increased in DN patients (*n* = 20/group). **D** Haematoxylin–eosin staining (HE) of renal biopsy specimens from DN patients and control participants. **E** Masson staining of renal biopsy specimens from DN patients and control participants. **F** Immunohistochemistry (IHC) of p-p65 in renal biopsy specimens of DN patients and control participants. **G** IHC of PTP1B in renal biopsy specimens of DN patients and control participants. **H**–**J** ELISA indicated that IL-1β, TNFα and IL-6 were increased in DN rats (*n* = 10/group). **K** HE of renal biopsy specimens of DN rats and control rats. **L** Masson staining of renal biopsy specimens of DN rats and control rats. **M** IHC of p-p65 in renal biopsy specimens of DN rats and control rats. **N** IHC of PTP1B in renal biopsy specimens of DN rats and control rats. (*p < 0.05, **p < 0.01, ***p < 0.001, ****p < 0.0001, *n* = 10/group).

The characteristics of DN patients are shown in Table 1. Overexpression of various inflammatory factors has been reported to play a crucial role in the renal reaction to inflammation and thus the pathogenesis of DN^7^. Therefore, we detected plasma inflammatory factor levels in control participants and DN patients. Compared with control participants, the plasma levels of inflammatory factors, including IL-1β, TNFα and IL-6, were increased in DN patients (Fig. 1A–C). With the development of inflammation in DN, the recruitment of fibroblasts ultimately results in renal injury and fibrosis^13^. HE staining of the renal biopsy specimens of DN patients showed thicker glomerular basement membranes and capillary occlusion (Fig. 1D). Masson trichrome staining of renal biopsy specimens of DN patients showed more blue staining, which represents collagen deposition and extensive interstitial fibrosis (Fig. 1E). Moreover, compared with control participants, p65 phosphorylation in glomerular endothelial cells was increased in DN patients (Fig. 1F). PTP1B regulates the expression of a series of cytokines and NF-κB activity^12^. Accordingly, we detected PTP1B expression and found that PTP1B expression in glomerular endothelial cells was higher in DN patients than in control participants (Fig. 1G). These data indicated that plasma inflammatory factor levels, p-p65 and PTP1B may participate in the occurrence and progression of DN.

**Table 1:** Characteristics of participants and rats in the control (con) and diabetic nephropathy (DN) groups.

Variables	con	DN	*P* value
*Human*			
Male (%)	65	60	0.744
Age (years)	53.7 ± 6.64	53.1 ± 9.05	0.812
BMI (kg/m2)	24.6 ± 3.03	24.5 ± 2.47	0.935
SBP (mmHg)	119.95 ± 10.06	144.05 ± 16.76	<0.0001
DBP (mmHg)	66.50 ± 5.66	77.50 ± 11.51	<0.0001
HbA1C (%)	5.2 ± 0.35	7.1 ± 1.2	<0.0001
FBG (mmol/l)	4.5 ± 0.65	8.2 ± 2.1	<0.0001
CREA (umol/l)	58.9 ± 7.2	175.3 ± 95.6	<0.0001
ALB (g/L)	43.5 ± 5.2	31.5 ± 6.3	<0.001
CCr (ml/min)	102.3 ± 10.3	55.6 ± 28.3	<0.0001
24hUTP (mg)	121.8 ± 23.1	3452.6 ± 2956.2	<0.0001
UA (µmol/L)	223.8 ± 42.3	389.5 ± 85.2	<0.0001
TP (g/L)	69.3 ± 9.5	52.1 ± 9.2	<0.001
**Variables**	**con**	**DM**	***P*** **value**
*Rats*			
FBG (mmol/l)	4.2 ± 0.8	21.5 ± 3.6	<0.0001
CREA (µmol/l)	23.5 ± 3.7	40.3 ± 10.2	<0.0001
UREA (mmol/l)	2.4 ± 0.3	6.5 ± 1.0	<0.0001
24hUTP (mg)	32.5 ± 5.2	369.1 ± 80.2	<0.0001

Data are presented as means ± SD. *BMI* body mass index, *SBP* systolic blood pressure, *DBP* diastolic blood pressure, *HbA1c* glycated haemoglobin, *FBS* fasting blood sugar, *CREA* creatinine, *ALB* albumin, *CCr* creatinine clearance, *24hUTP* 24-hour urinary protein quantity, *UA* uric acid, *TP* total protein.

The characteristics of the DN rats are shown in Table 1. Consistent with the observations above, plasma inflammatory factor levels were augmented in DN rats (Fig. 1H–J). Moreover, HE (Fig. 1K) and Masson trichrome staining (Fig. 1L) indicated that glomerular injury and fibrosis were present in DN rats. Furthermore, the levels of p65 phosphorylation and PTP1B expression in kidney tissues were higher in DN rats than in the control group (Fig. 1M, N). Protein and/or mRNA levels of p-p65 and/or PTP1B in renal and aortic tissues were also higher in DN rats than in the control group (Supplementary Fig. 1).

### High glucose treatment induces inflammatory factor levels via upregulation of PTP1B expression in endothelial cells

**Fig. 2:**
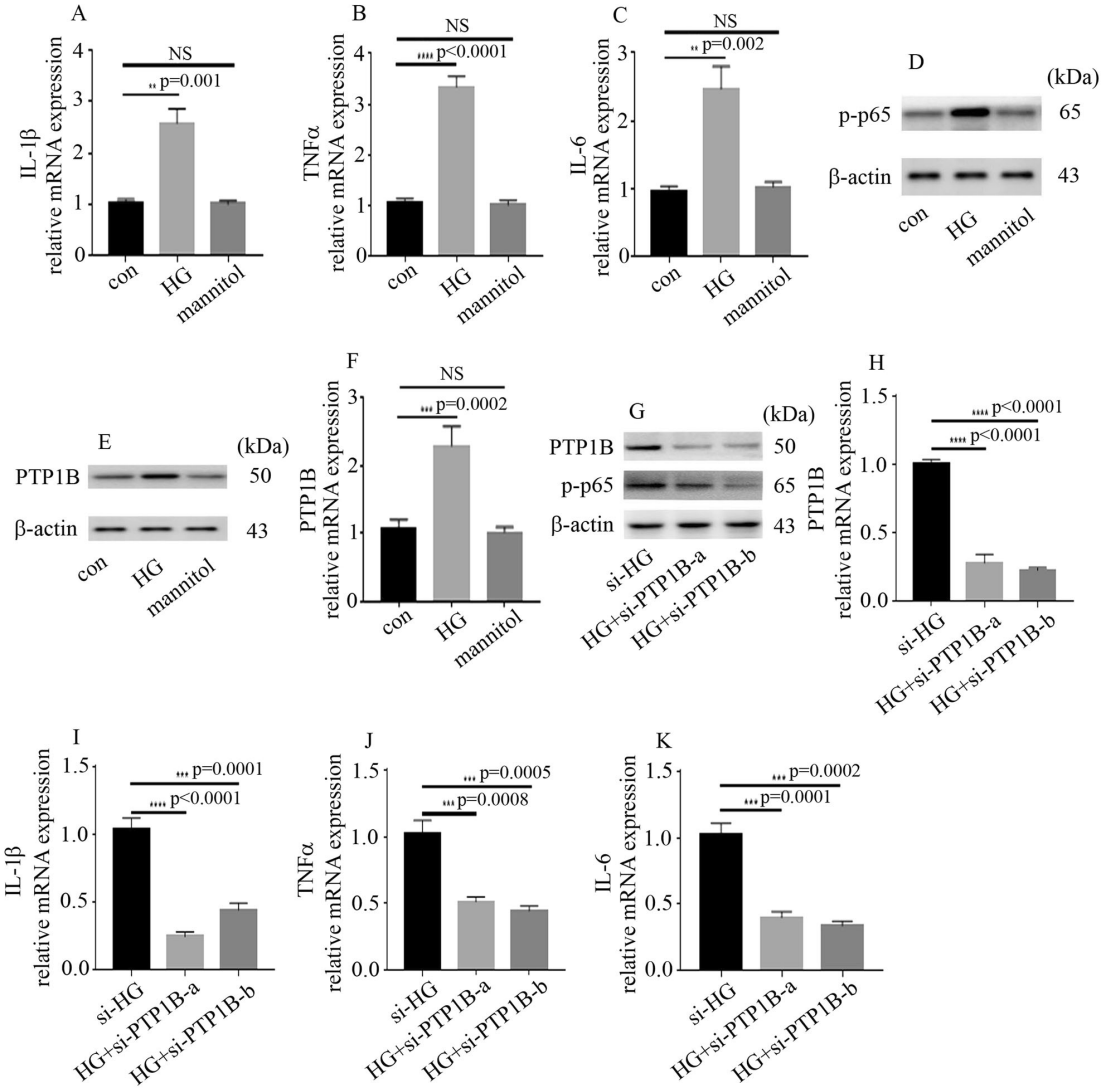
High glucose treatment induces elevated inflammatory factor levels via upregulation of PTP1B expression in endothelial cells. **A**–**C** qPCR analysis of mRNA expression of IL-1β, TNFα and IL-6. **D** western blot analysis of p-p65 levels in HUVECs. **E** western blot analysis of PTP1B levels in HUVECs. **F** qPCR analysis of mRNA expression of PTP1B. **G** Western blot analysis of PTP1B and p-p65 levels in HUVECs. **H**–**K** qPCR analysis of mRNA expression of PTP1B, IL-1β, TNFα and IL-6. (*p < 0.05, **p < 0.01, ***p < 0.001, ****p < 0.0001, *n* = 5/group).

The potential regulatory mechanism underlying the link between PTP1B and plasma inflammatory factor levels in DN patients and rats was explored using HUVECs. To determine whether high glucose increases endothelial inflammatory factor levels and PTP1B expression in HUVECs, cells were cultured in normal-glucose DMEM (con, 5 mM, 6 days) or high-glucose DMEM (HG, 25 mM, 6 days). High glucose treatment augmented inflammatory factor levels (Fig. 2A–C) and p65 phosphorylation (Fig. 2D) in HUVECs. These data indicated that high glucose treatment-induced endothelial inflammatory factor levels in HUVECs. PTP1B regulates the expression of a series of cytokines and NF-κB activity^12^; therefore, we sought to detect PTP1B expression in HUVECs. High glucose treatment increased PTP1B protein (Fig. 2E) and mRNA (Fig. 2F) levels in HUVECs. Mannitol had no effect on cellular inflammatory factor levels or PTP1B expression (Fig. 2A–F). To further illustrate whether PTP1B is involved in high glucose-induced p65 phosphorylation and inflammatory factor levels in HUVECs, two independent siRNAs against PTP1B were used. The effects of si-PTP1B were confirmed by western blotting (Fig. 2G) and qPCR (Fig. 2H). si-PTP1B treatment inhibited high glucose-induced p65 phosphorylation and cellular inflammatory factor levels in HUVECs (Fig. 2G, I–K). These data indicated that PTP1B positively regulates endothelial inflammatory factor levels in hyperglycaemic HUVECs.

### CREB participates in high glucose-induced p65 phosphorylation and inflammatory factor levels via negative regulation of PTP1B expression in HUVECs

**Fig. 3:**
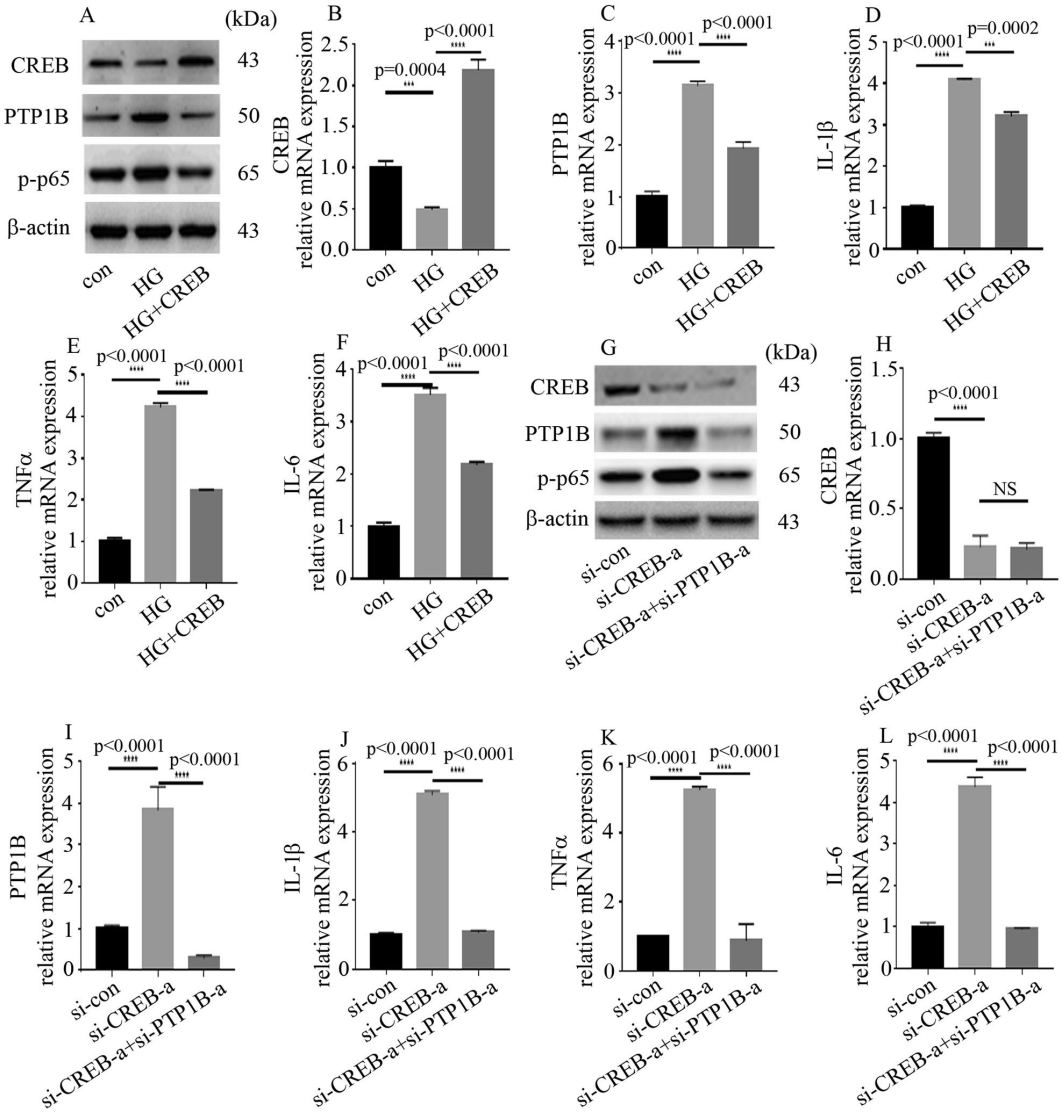
CREB participates in high glucose-induced p65 phosphorylation and inflammatory factor levels via negative regulation of PTP1B expression in endothelial cells. **A** Western blot analysis of CREB, PTP1B and p-p65 levels in HUVECs. **B**–**F** qPCR analysis of mRNA expression of CREB, PTP1B, IL-1β, TNFα and IL-6. **G** western blot analysis of CREB, PTP1B and p-p65 levels in HUVECs. **H**–**L** qPCR analysis of mRNA expression of CREB, PTP1B, IL-1β, TNFα and IL-6. (*p < 0.05, **p < 0.01, ***p < 0.001, ****p < 0.0001, *n* = 5/group).

Previous studies have indicated that CREB is important in the maintenance of a healthy endothelium^15–18^. High glucose treatment decreased both protein and mRNA levels of CREB (Supplementary Fig. 2A, B). To explore the effect of CREB on high glucose-induced PTP1B expression and cellular inflammatory factor levels, both loss-of-function and gain-of-function approaches were employed. The effect of CREB overexpression in hyperglycaemic HUVECs was verified by western blot (Fig. 3A) and qPCR (Fig. 3B). CREB overexpression reversed high glucose-induced PTP1B expression (Fig. 3A, C), p65 phosphorylation (Fig. 3A) and endothelial inflammatory factor levels (Fig. 3D–F) in HUVECs. Moreover, the effect of si-CREB was similar to that of high glucose treatment (Supplementary Fig. 2C–H). To determine whether the effects of si-CREB were due to increased PTP1B expression, we silenced PTP1B in HUVECs in which CREB was downregulated (Fig. 3G–I). Our data indicated that PTP1B silencing counteracts CREB silencing-induced p65 phosphorylation (Fig. 3G) and endothelial inflammatory factors in HUVECs (Fig. 3J–L). These data indicated that CREB downregulation increases p65 phosphorylation and endothelial inflammatory factor levels by augmenting PTP1B expression.

### CREB interacts with KMT5A

**Fig. 4:**
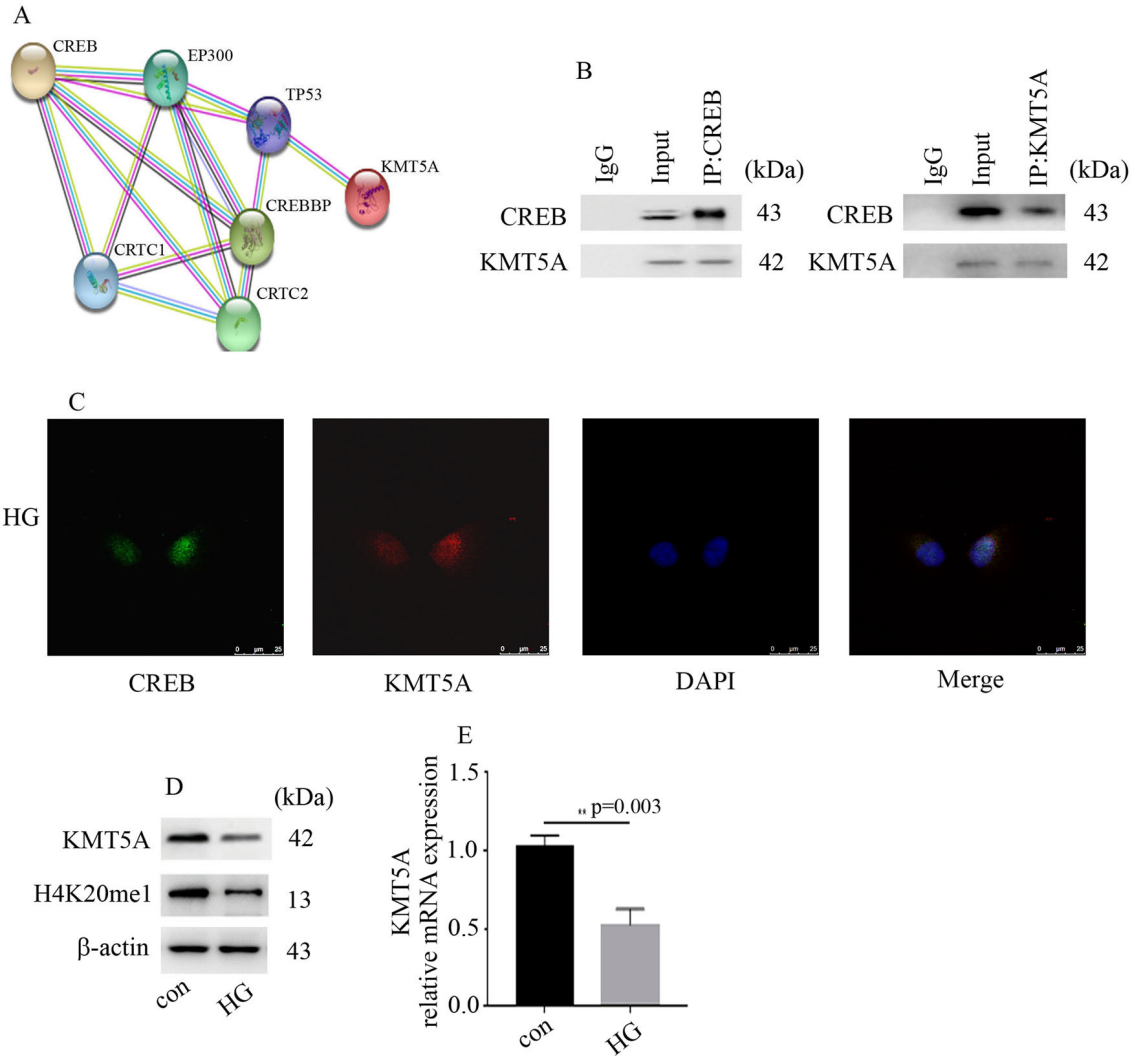
CREB interacts with KMT5A. **A** Several proteins that interact with CREB are shown (https://inbio-discover.intomics.com/map.html#search). **B** The association between CREB and KMT5A in HUVECs was confirmed by CoIP. **C** Colocalization of CREB and KMT5A in HUVECs was assessed by confocal microscopy. **D** western blot analysis of KMT5A and H4K20me1 levels in HUVECs. **E** qPCR analysis of mRNA expression of KMT5A (*p < 0.05, **p < 0.01, ***p < 0.001, ****p < 0.0001, *n* = 5/group).

To uncover the potential regulatory mechanism by which CREB modulates PTP1B expression and endothelial inflammation in HUVECs, bioinformatics was employed to predict the proteins that interact with CREB. Figure 4A shows that many proteins interact with CREB (https://inbio-discover.intomics.com/map.html#search). We previously demonstrated that KMT5A suppression contributes to high glucose-induced endothelial injury^20–24^. The interaction between CREB and KMT5A in HUVECs was confirmed by CoIP experiments (Fig. 4B). Double immunofluorescent staining revealed colocalization of CREB and KMT5A in the nucleus in hyperglycaemic HUVECs (Fig. 4C). Furthermore, high glucose treatment inhibited KMT5A protein and mRNA expression in HUVECs (Fig. 4D, E). Consistent with these observations, levels of H4K20me1, a downstream target of KMT5A, were reduced by high glucose treatment (Fig. 4D).

### KMT5A suppression participates in high glucose-mediated endothelial inflammation via augmentation of PTP1B expression in HUVECs

**Fig. 5:**
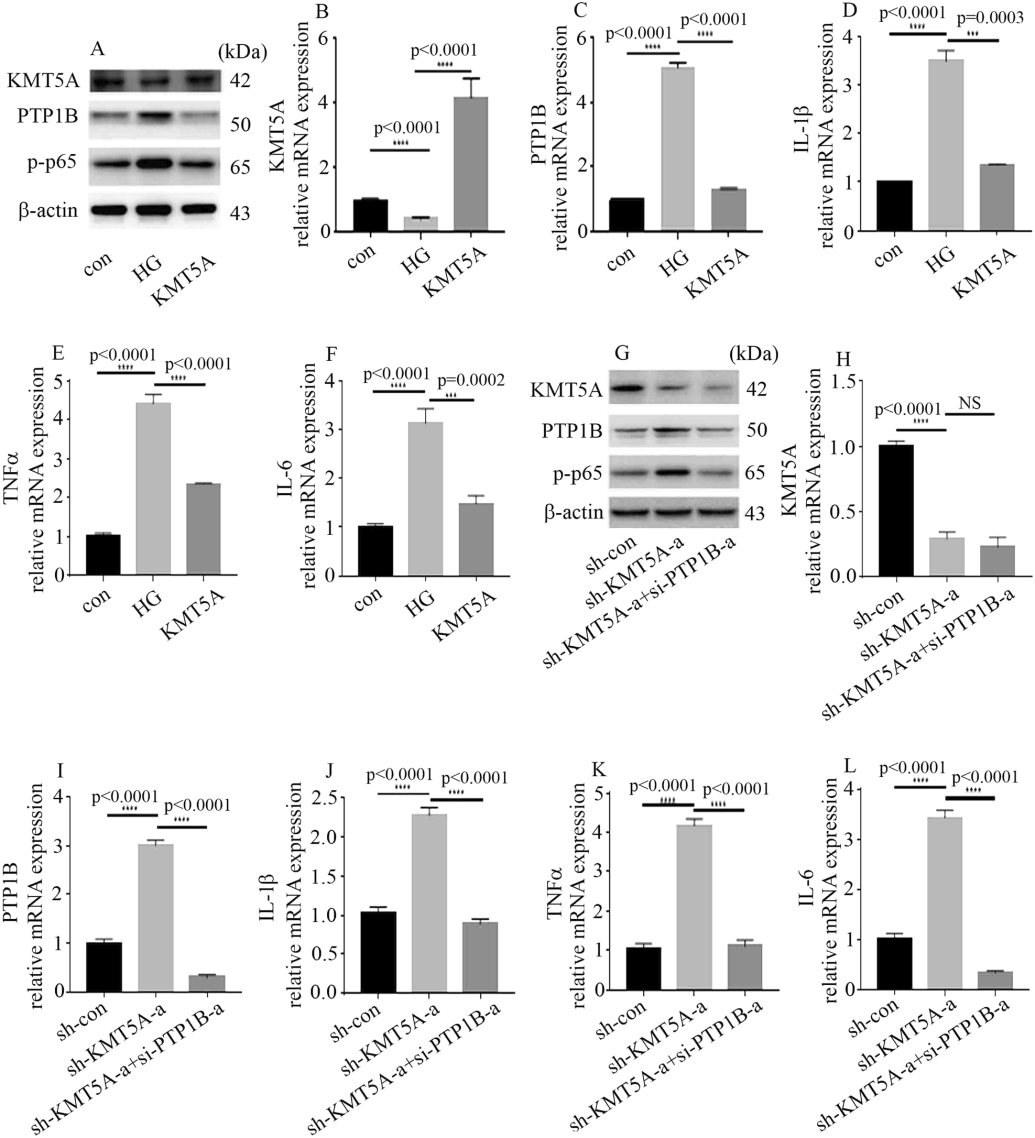
KMT5A suppression participates in high glucose-mediated endothelial inflammation by augmenting PTP1B expression in endothelial cells. **A** Western blot analysis of KMT5A, PTP1B and p-p65 levels in HUVECs. **B**–**F** qPCR analysis of mRNA expression of KMT5A, PTP1B, IL-1β, TNFα, and IL-6. **G** Western blot analysis of KMT5A, PTP1B and p-p65 levels in HUVECs. **H**–**L** qPCR analysis of mRNA expression of KMT5A, PTP1B, IL-1β, TNFα, and IL-6. (*p < 0.05, **p < 0.01, ***p < 0.001, ****p < 0.0001, *n* = 5/group).

To determine the effect of KMT5A on high glucose-induced PTP1B expression, p65 phosphorylation and inflammatory factor levels in HUVECs, both loss-of-function and gain-of-function approaches were used. The effect of KMT5A overexpression in hyperglycaemic HUVECs was verified by western blot (Fig. 5A) and qPCR (Fig. 5B). KMT5A overexpression reversed high glucose-induced PTP1B expression (Fig. 5A, C), p65 phosphorylation (Fig. 5A) and endothelial inflammatory factor levels (Fig. 5D–F) in HUVECs. Moreover, the effect of sh-KMT5A was similar to that of high glucose treatment (Supplementary Fig. 3A–F). To determine whether the effects of sh-KMT5A were the result of increased PTP1B expression, we silenced PTP1B in HUVECs in which KMT5A was downregulated (Fig. 5G–I). PTP1B silencing counteracted KMT5A downregulation-induced p65 phosphorylation and endothelial inflammatory factor levels in HUVECs (Fig. 5G, J–L). These data indicated that KMT5A downregulation participates in high glucose-induced p65 phosphorylation and endothelial inflammatory factor levels by augmenting PTP1B expression.

### CREB cooperates with KMT5A to regulate PTP1B transcriptional activity in HUVECs

**Fig. 6:**
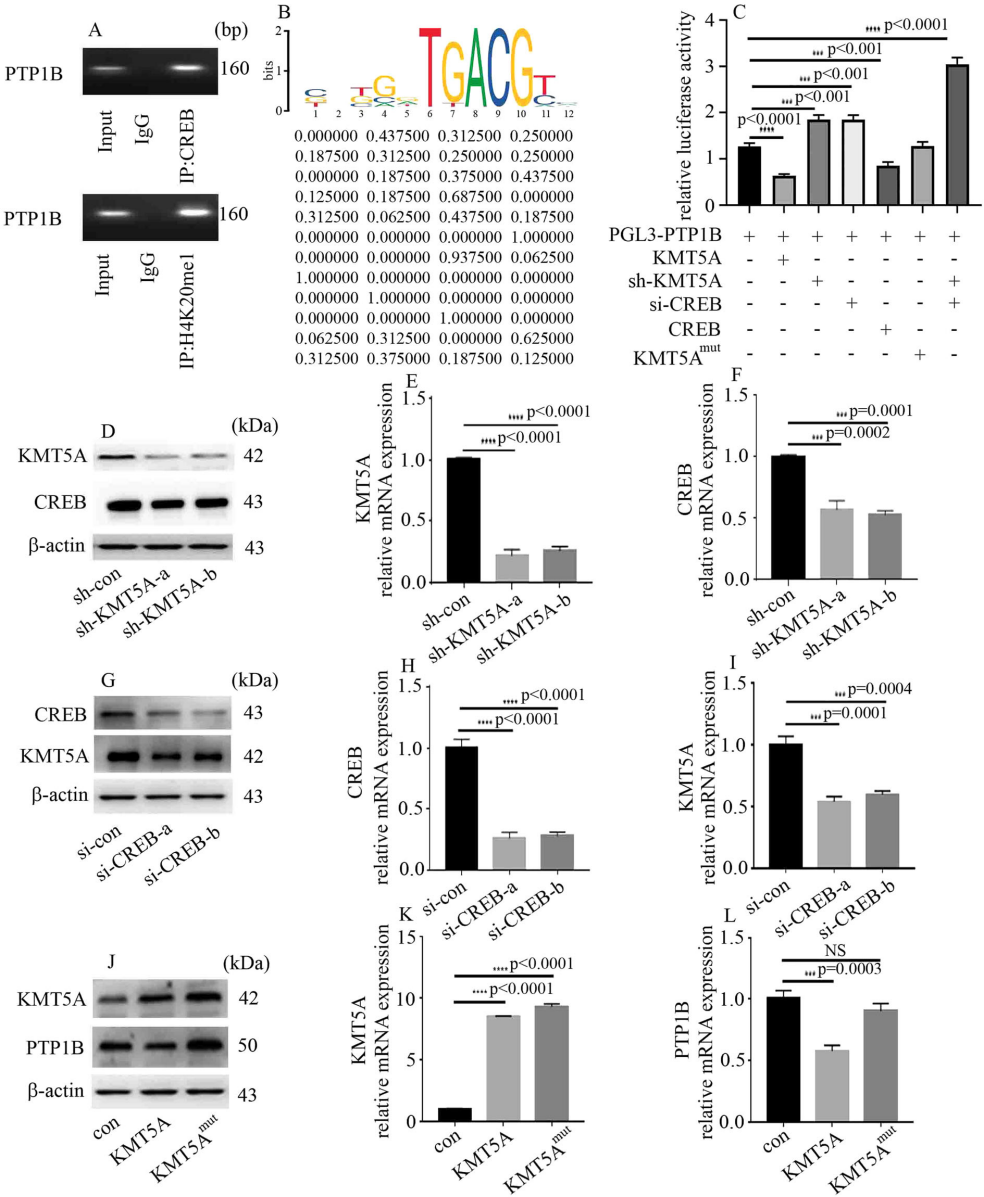
CREB interacts with KMT5A to regulate PTP1B transcriptional activity in endothelial cells. **A** CREB and H4K20me1 were enriched at the PTP1B promoter region. **B** The putative CREB binding site of PTP1B. **C** PTP1B promoter activity was determined by luciferase reporter assays. **D** western blot analysis of KMT5A and CREB levels in HUVECs. **E**, **F** qPCR analysis of mRNA expression of KMT5A and CREB. **G** western blot analysis of KMT5A and CREB levels in HUVECs. **H**, **I** qPCR analysis of mRNA expression of KMT5A and CREB. **J** western blot analysis of KMT5A and PTP1B levels in HUVECs. **K**, **L** qPCR analysis of mRNA expression of KMT5A and PTP1B. (*p < 0.05, **p < 0.01, ***p < 0.001, ****p < 0.0001, *n* = 5/group).

To explore whether PTP1B is directly targeted by CREB and KMT5A, we detected the genome-wide distribution of CREB and H4K20me1 in HUVECs by a ChIP assay. The results demonstrated that CREB and H4K20me1 were both enriched in the PTP1B promoter region (Fig. 6A). The putative CREB-binding site is shown in Fig. 6B. The motif logo and position weight matrix are shown in the upper and lower panels, respectively (Fig. 6B). KMT5A overexpression and CREB overexpression both reduced PTP1B promoter activity (Fig. 6C). sh-KMT5A not only increased PTP1B promoter activity but also increased the positive effect of si-CREB on PTP1B promoter activity (Fig. 6C). These data demonstrated that CREB cooperates with KMT5A to modulate PTP1B promoter activity in hyperglycaemic HUVECs. Furthermore, sh-KMT5A inhibited CREB expression in HUVECs (Fig. 6D–F). Consistently, si-CREB decreased KMT5A expression in HUVECs (Fig. 6G–I). These data indicated that KMT5A and CREB positively modulate each other in HUVECs. Furthermore, KMT5A overexpression attenuated PTP1B expression, whereas the mutant KMT5A^R259G^ did not affect PTP1B expression (Fig. 6J–L). These data demonstrated that KMT5A-mediated H4K20me1 is necessary to modulate PTP1B expression in HUVECs.

### Glomerular CREB and KMT5A expression are decreased in DN patients and rats

**Fig. 7:**
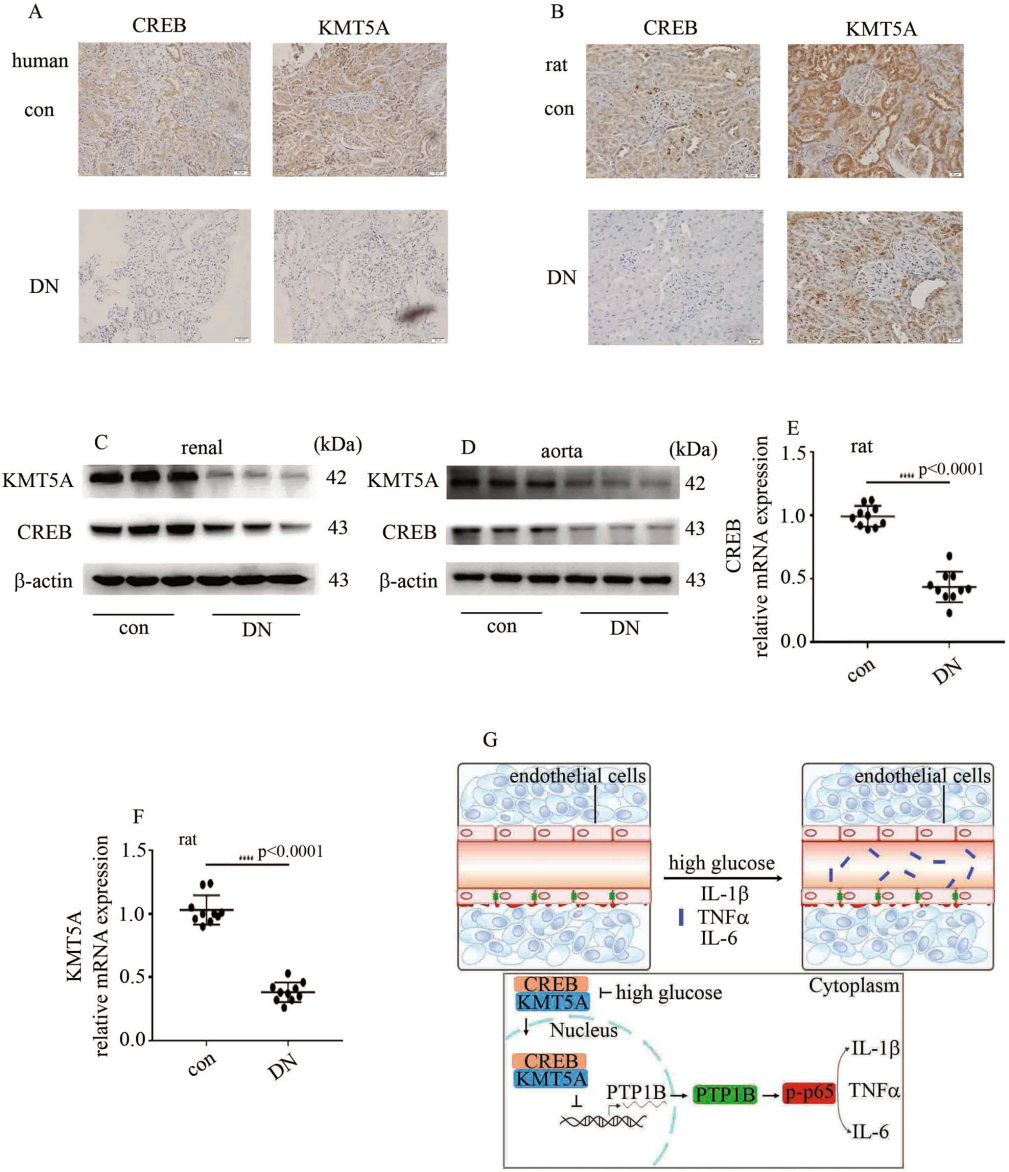
Verification of high glucose-mediated decreases in KMT5A and CREB in DN patients and rats. **A** Immunostaining of CREB and KMT5A in renal biopsy specimens of DN patients and control participants (*n* = 20/group). **B** Immunostaining of CREB and KMT5A in renal biopsy specimens of DN rats and control rats (*n* = 10/group). **C** Protein expression of CREB and KMT5A in the renal tissues of control rats and DN rats was assessed by western blot. **D** Protein expression of CREB and KMT5A in the aorta tissues of control rats and DN rats was assessed by western blot. **E**, **F** mRNA expression of CREB and KMT5A in aorta tissues of DN rats and control rats was assessed by qPCR (*n* = 10/group). **G** Schematic representation of the working model. (*p < 0.05, **p < 0.01, ***p < 0.001, ****p < 0.0001, *n* = 10/group).

To explore whether the protein and/or mRNA expression of CREB and KMT5A in DN patients and rats were consistent with the results obtained in vitro, we assessed CREB and KMT5A expression in kidney tissues and/or aortic tissues of DN patients and rats. CREB and KMT5A expression were reduced in glomerular endothelial cells of DN patients (Fig. 7A). Consistent with this observation, CREB and KMT5A expression was also decreased in glomerular endothelial cells of DN rats (Fig. 7B). Moreover, the protein and/or mRNA levels of CREB and KMT5A in renal and aortic tissues were lower in DN rats than in control rats (Fig. 7C–F). In conclusion, our study showed that CREB cooperates with KMT5A to regulate PTP1B transcription, thus mediating endothelial inflammation in glomerular endothelial cells of DN patients and rats (Fig. 7G).

## Discussion

Our study indicated that high glucose, by augmenting PTP1B expression, participates in the modulation of endothelial inflammatory factor levels, thus mediating glomerular endothelial cell injury and the occurrence of DN. Moreover, exposure to high glucose reduced CREB and KMT5A expression, and CREB and H4K20me1 were both enriched in the PTP1B promoter region. Mechanistic studies showed that CREB cooperates with KMT5A to regulate PTP1B transcriptional activity, thus augmenting endothelial inflammatory factor levels in hyperglycaemic HUVECs.

The activation of inflammatory pathways is reported to be a major contributor to DN pathogenesis^7^. Inflammatory cytokines, including IL-1β, TNFα and IL-6, participate in DN progression^8,9^. Moreover, NF-κB activation plays a crucial role in inflammatory factor expression in DN^10,11^. With the development of inflammation in DN, the recruitment of fibroblasts ultimately results in renal fibrosis and injury^13^. PTP1B modulates the expression of a series of cytokines and NF-κB activity^12^. Moreover, podocyte-specific PTP1B-overexpressing mice develop spontaneous proteinuria and renal injury^25^, and PTP1B deficiency in podocytes mitigates hyperglycemia-induced renal injury^26^. In the present study, PTP1B expression was increased in glomerular endothelial cells of DN patients and rats (Fig. 1G, N; Supplementary Fig. 1A–C). In an in vitro study, inhibition of PTP1B expression reversed high glucose-induced p65 phosphorylation, thus attenuating endothelial inflammatory factor levels in HUVECs (Fig. 2G, I–K). Our data may indicate that inhibition of PTP1B expression in glomerular endothelial cells attenuates hyperglycemia-induced renal injury.

CREB is widely expressed and is important in modulating cellular metabolism, cell cycle progression, survival, and responses to extracellular stimuli^14^. Recent studies have demonstrated that CREB is important in the maintenance of a healthy endothelium^15–18^. Vascular CREB levels are decreased in in vivo models of hypertension, atherosclerosis and insulin resistance^27^, and targeted cardiac expression of dominant-negative CREB augments oxidative stress, mitochondrial dysfunction and mortality in vivo^28^. These studies indicate that CREB plays a crucial role in the modulation of diverse protective genes. In the present study, CREB overexpression inhibited high glucose-induced PTP1B expression and p65 phosphorylation, thus attenuating inflammatory factor levels in HUVECs (Fig. 3A, C–F). Moreover, CREB was enriched in the PTP1B promoter region (Fig. 6A), and si-PTP1B reversed CREB knockdown-induced p65 phosphorylation and inflammatory factor levels (Fig. 3G, J–L). These data indicated that CREB overexpression inhibits high glucose-induced p65 phosphorylation and inflammatory factor levels via the suppression of PTP1B. In vivo, CREB expression was inhibited in DN patients and rats (Fig. 7A–E). These results may indicate that CREB downregulation increases p65 phosphorylation and inflammatory factor levels via upregulation of PTP1B to contribute to the occurrence and progression of DN.

We previously demonstrated that KMT5A participates in high glucose-induced endothelial adhesion molecule expression^20^, proinflammatory enzyme and proinflammatory cytokine production^21^, antioxidant imbalance^22^, NOD-like receptor pyrin domain 3 inflammasome activation^23^ and endothelial adhesion molecule expression^24^ in HUVECs to mediate vascular endothelial injury^20–24^. In the present study, KMT5A overexpression inhibited high glucose-induced PTP1B and p65 phosphorylation, thus alleviating high glucose-induced inflammatory factor levels (Fig. 5A, C–F). Moreover, H4K20me1, a downstream target of KMT5A, was enriched in the PTP1B promoter region (Fig. 6A), and si-PTP1B reversed KMT5A knockdown-induced p65 phosphorylation and inflammatory factor levels (Fig. 5G, I–L). These data indicated that KMT5A overexpression inhibits high glucose-induced p65 phosphorylation and inflammatory factor levels via suppression of PTP1B. The in vivo study showed that KMT5A expression was inhibited in DN patients and rats (Fig. 7A–D, F). These results may indicate that KMT5A downregulation increases p65 phosphorylation and inflammatory factor levels via upregulation of PTP1B to participate in the occurrence and progression of DN.

The transcriptional activity of CREB is modulated by epigenetic modifications^29^. The present study revealed an association between CREB and KMT5A (Fig. 4A–C). Moreover, CREB and H4K20me1 both occupy the PTP1B promoter region (Fig. 6A), and sh-KMT5A increased the positive effect of si-CREB on PTP1B promoter activity (Fig. 6C). These results demonstrated that CREB cooperates with KMT5A to regulate PTP1B transcription, thus increasing p65 phosphorylation and inflammatory factor levels in hyperglycaemic HUVECs. Furthermore, KMT5A overexpression inhibited PTP1B expression, whereas the KMT5A mutant KMT5A^R259G^ did not affect PTP1B expression (Fig. 6J–L). These data indicated that KMT5A-mediated H4K20me1 participates in the regulation of PTP1B expression.

This study has some limitations. First, whether CREB associates with KMT5A directly or indirectly deserves further research. Second, this study was performed using HUVECs, and our findings need to be validated using other primary endothelial cell models. Third, the mechanistic studies were mainly carried out in HUVECs, and the results should be confirmed in an in vivo study. Fourth, the mechanism by which KMT5A and CREB positively regulate each other warrants further research.

In conclusion, our data indicated that CREB and KMT5A expression were decreased and PTP1B expression and p65 phosphorylation were increased in glomerular endothelial cells of DN patients and rats. Moreover, plasma inflammatory factor levels were increased in DN patients and rats. The present study also demonstrated that high glucose increased PTP1B expression in hyperglycaemic HUVECs, thus inducing inflammatory factor levels in HUVECs. Moreover, high glucose inhibited CREB and KMT5A expression. Furthermore, CREB cooperated with KMT5A to modulate PTP1B expression, p65 phosphorylation and inflammatory factor levels, thus participating in the occurrence and progression of DN.

## Material and methods

### Cell culture and reagents

HUVECs were obtained from American Type Culture Collection (ATCC; Manassas, USA) and cultured in Dulbecco’s modified Eagle medium (DMEM) with 5 mM glucose supplemented with 10% foetal bovine serum and penicillin/streptomycin (100 g/ml) at 37 °C in a humidified 5% carbon dioxide incubator. For high glucose treatment, cells were washed with phosphate-buffered saline three times to remove the complete medium and further cultured in DMEM with 25 mM glucose, 10% foetal bovine serum and penicillin/streptomycin (100 g/ml) for 6 days. Glucose (5 mM) plus mannitol (20 mM) was used as an osmotic control.

### Subjects

Twenty biopsy-proven DN patients (type 2 diabetes) and twenty control participants (renal cancer patients with normal renal function) recruited consecutively from Huzhou Central Hospital were included. The study complied with the Declaration of Helsinki and was approved by the Ethics Committee of Huzhou Central Hospital (licence No. 20191209-01). Written informed consent was obtained from all subjects. The exclusion criteria were an advanced liver disease, valvular heart disease, severe heart failure, stroke, atrial fibrillation, peripheral arterial disease and other vascular diseases.

### Rat model of DN and samples

Male Sprague–Dawley rats weighing 180–200 g were purchased from Shanghai SLAC Laboratories. The present study complied with the Guide for the Care and Use of Laboratory Animals of Shanghai Medical College at Fudan University and was performed according to the Institutional Guidelines for Animal Research and the Guide for the Care and Use of Laboratory Animals published by the US NIH (2011). The rats were randomly allocated to the control group (con, *n* = 10) and DN group (DN, *n* = 10). The rats in the control group were injected intraperitoneally with citrate buffer (0.1 M, pH 4.5). Rats in the DN group were fed a high sugar-fat diet (67% basic feed, 10% lard, 20% sugar, 2.5% cholesterol, and 0.5% sodium cholate) for 2 weeks before receiving a single intraperitoneal injection of streptozotocin (STZ, 50 mg/kg); the rats were then returned to standard laboratory chow for 6 weeks. Hyperglycaemia in rats was diagnosed by detecting blood glucose via tail-neck blood sampling 3 days after STZ injection. One day before euthanasia, urine was collected for 24 h, and albumin levels were detected with an enhanced BCA protein assay kit (Beyotime, Shanghai, China). Intraperitoneal administration of thiopental sodium (40 mg/kg) was used for euthanasia of all rats. Blood samples were obtained by cardiac puncture and collected in EDTA vacutainer tubes. Plasma samples and urine specimens were frozen at −80 °C until analysis. Serum creatinine levels and serum urea nitrogen (BUN) levels were detected with a creatinine assay kit (Jiancheng Bio, Nanjing, China) and BUN assay kit (Jiancheng Bio, Nanjing, China), respectively.

### Haematoxylin–eosin staining (HE), Masson staining and immunohistochemistry (IHC)

Standard IHC procedures were employed with anti-PTP1B (ProteinTech, Wuhan, China, 11334-1-AP), anti-p-p65 (Signalway antibody, Maryland, #11014), anti-KMT5A (ProteinTech, Wuhan, China, 14063-1-AP) and anti-CREB (Cell Signalling Technology, Danvers, MA, #9197) antibodies. The tissues were embedded in paraffin and then processed for staining with haematoxylin and eosin (HE) to detect histopathological abnormalities. Masson staining was used to determine the severity of fibrosis according to the kit’s instructions (Solarbio, Beijing, China).

### Western blot analysis

Whole cells were washed with ice-cold phosphate-buffered saline, harvested, and resuspended in lysis buffer (Cell Signalling Technology, Danvers, MA). Protein samples were boiled for 10 min at 100 °C in sample loading buffer. Equal amounts of protein (50 μg) from different groups of HUVECs were separated by 8–10% sodium dodecyl sulfate-polyacrylamide gel electrophoresis and transferred to PVDF membranes (Millipore, Billerica, USA). Membranes were blocked with 5% skimmed milk solution for 1 h, and then all membranes were incubated with primary antibodies at 4 °C for 12 h. The primary antibodies used in the present study were as follows: monoclonal antibodies against β-actin (ProteinTech, Wuhan, China, 60004-1-Ig), KMT5A (ProteinTech, Wuhan, China, 14063-1-AP), CREB (Cell Signalling Technology, Danvers, MA, #9197), PTP1B (ProteinTech, Wuhan, China, 11334-1-AP), p-p65 (Signalway antibody, Maryland, #11014) and H4K20me1 (Abcam, Cambridge, UK, ab9051). After washing the membranes with PBST 5 times, an HRP-conjugated secondary antibody was added for 1 h at room temperature. Then, the membranes were washed five times with PBST, and the protein signal was detected by an ECL system.

### RNA extraction and quantitative real-time PCR (qPCR)

Total RNA was extracted using TRIzol (Invitrogen, Grand Island, NY, USA) and used to synthesise complementary DNA (cDNA) with PrimeScript RT reagent (TaKaRa) according to the manufacturer’s instructions. qPCR was then performed using Hieff UNICON® qPCR TaqMan Probe Master Mix (Yeasen, Shanghai, China) in an ABI7500 Real-Time PCR system (Applied Biosystems). The primers used in the present study are shown in Supplementary Table 1.

### Co-immunoprecipitation (CoIP)

Whole-cell protein lysates were extracted with cell lysis buffer containing PMSF (Beyotime Biotechnology, Shanghai). For endogenous IP, 30 μl of lysate was incubated with the corresponding primary antibodies and 50 μl of protein A/G Dynabeads (Thermo Fisher, USA) at 4 °C for 12 h. Then, 10 μl of the input, IgG and IP fractions were analysed by western blotting.

### Immunofluorescence (IF) staining

HUVECs were plated onto glass slides with the corresponding treatment. After fixing with 4% paraformaldehyde, the cells were permeabilized with 0.3% Triton X-100 for 5 min and then blocked for 1 h at room temperature. The cells were incubated with anti-KMT5A (Proteintech, Wuhan, China, 14063-1-AP) and anti-CREB (Cell Signalling Technology, Danvers, MA, #9197) antibodies at 4 °C for 12 h. 4,6-Diamidino-2-phenylindole (DAPI) was used for nuclear staining. Images were acquired with a confocal Leica fluorescence microscope.

### siRNA, shRNA and KMT5A mutant treatments

HUVECs were transfected with sh-KMT5A, a mutant KMT5A^R259G^ plasmid^23^, si-CREB and si-PTP1B using Lipofectamine 3000 (Invitrogen, USA). The sequences of sh-KMT5A (Biotend, Shanghai, China) were shRNA-a, 5′-CAACAGAATCGCAAACTTA-3′, and shRNA-b, 5′-CAACAGAATCGCAAACTTA-3′. The sequences of si-CREB (Biotend, Shanghai, China) were siRNA-a, 5′-AACCAAGTTGTTGTTCAAGCT-3′, and siRNA-b, 5′-AGUAAAGGUCCUUAAGUGCTT-3′. The sequences of si-PTP1B (Biotend, Shanghai, China) were siRNA-a, 5′-AUAGGUACAGAGACGUCAGUU-3′, and siRNA-b, 5′-CCAAGAAACUCGAGAGAUC-3′.

### Chromatin immunoprecipitation (ChIP) assay

ChIP assays were performed with a Simple ChIP Plus Sonication Chromatin IP Kit (Cell Signalling Technology, MA) according to the manufacturer’s instructions. Briefly, the cells (1 × 10^7^) were fixed with 1% formaldehyde for 10 min at room temperature to cross-link the DNA and the proteins. The cross-linking reaction was terminated by adding glycine. A Microson XL ultrasonic cell disruptor XL (Misonix) was used to shear the chromatin. Ten microlitres of the sonicated solution was removed as an input control. The remaining sonicated solution was incubated with an anti-CREB antibody (Cell Signalling Technology, Danvers, MA, #9197), anti-H4K20me1 antibody (Abcam, Cambridge, UK, ab9051) or negative control IgG at 4 °C for 12 h. The immunoprecipitates were bound to protein G magnetic beads, and DNA-protein cross-linking was terminated by incubation at 65 °C for 2 h. After purification, the enriched DNA sequences were detected by PCR. The PTP1B oligonucleotide primer sequences were forward, 5′-AGCCTCCCAAGTAGCTGGGA-3′, and reverse 5′GCACTTTGGGAGACCGACGC-3′.

### Dual-luciferase assay

The effects of KMT5A and CREB on the activity of the PTP1B promoter were assessed using a Promega Dual-luciferase Assay Kit (Madison, WI, United States). The PTP1B promoter was amplified and ligated into the pGL3-basic vector to create the pGL3-PTP1B construct. HUVECs were then transfected with the pGL3-PTP1B plasmid along with a Renilla luciferase vector. The effects of KMT5A and CREB on PTP1B promoter activity were evaluated using a dual-luciferase assay kit.

### Statistical analysis

The sample sizes of animals and HUVECs were determined by assessing high glucose- or hyperglycemia-induced PTP1B mRNA levels in pilot experiments. We anticipated that statistical significance could be achieved with a sample size of 10 in in vivo experiments and 5 in in vitro experiments. The data were acquired from at least 5 experiments performed separately, and the results are shown as the mean ± SD. Two-tailed unpaired *t* tests or one-way ANOVAs with GraphPad Prism Version 7.0 (GraphPad Software, San Diego, CA) were used to compare the groups. *P* < 0.05 was considered significant.

## Supplementary information

Supplementary figure 1

Supplementary figure 2

Supplementary figure 3

supplementary figure legneds

Supplementary Table 1

## Data Availability

The datasets used and/or analysed during the current study are available from the corresponding author on reasonable request.

## References

[1] Packham D (2012). Relative incidence of ESRD versus cardiovascular mortality in proteinuric type 2 diabetes and nephropathy: results from the DIAMETRIC (Diabetes Mellitus Treatment for Renal Insufficiency Consortium) database. Am. J. Kidney Dis..

[2] Tomino Y, Gohda T (2015). The prevalence and management of diabetic nephropathy in Asia. Kidney Dis..

[3] Ng K (2016). Results and lessons from the spironolactone to prevent cardiovascular events in early stage chronic kidney disease (STOP-CKD) randomized controlled trial. BMJ Open.

[4] Xue R (2017). Mechanistic insight and management of diabetic nephropathy: recent progress and future perspective. J. Diabetes Res..

[5] Rao J (2017). The RhoA/ROCK pathway ameliorates adhesion and inflammatory infiltration induced by AGEs in glomerular endothelial cells. Sci. Rep..

[6] Pedrinelli R, Dell’Omo G, Penno G, Mariani M (2001). Non-diabetic microalbuminuria, endothelial dysfunction and cardiovascular disease. Vasc. Med..

[7] Moreno J (2018). Targeting inflammation in diabetic nephropathy: a tale of hope. Expert Opin. Investig. Drugs.

[8] Garrido W (2019). Blockade of the adenosine A3 receptor attenuates caspase 1 activation in renal tubule epithelial cells and decreases interleukins IL-1β and IL-18 in diabetic rats. Int. J. Mol. Sci..

[9] Niewczas M (2009). Serum concentrations of markers of TNF alpha and Fas-mediated pathways and renal function in nonproteinuric patients with type 1 diabetes. Clin. J. Am. Soc. Nephrol..

[10] Cooper M (2001). Interaction of metabolic and haemodynamic factors in mediating experimental diabetic nephropathy. Diabetologia.

[11] Luo C (2015). Kaempferol alleviates insulin resistance via hepatic IKK/NF-κB signal in type 2 diabetic rats. Int. Immunopharmacol..

[12] Yang L (2020). PTP1B promotes macrophage activation by regulating the NF-κB pathway in alcoholic liver injury. Toxicol. Lett..

[13] Liu Y (2006). Renal fibrosis, new insights into the pathogenesis and therapeutics. Kidney Int..

[14] Shaywitz A, Greenberg M (1999). CREB: a stimulus-induced transcription factor activated by a diverse array of extracellular signals. Annu. Rev. Biochem..

[15] Suehiro J, Hamakubo T, Kodama T, Aird W, Minami T (2010). Vascular endothelial growth factor activation of endothelial cells is mediated by early growth response-3. Blood.

[16] Peshavariya H (2014). Prostacyclin signalling boosts NADPH oxidase 4 in the endothelium promoting cytoprotection and angiogenesis. Antioxid. Redox Signal..

[17] Bussolati B (2004). Bifunctional role for VEGF-induced heme oxygenase-1 in vivo: induction of angiogenesis and inhibition of leukocytic infiltration. Blood.

[18] Francesco L (2009). Induction of prostacyclin by steady laminar shear stress suppresses tumor necrosis factor-a biosynthesis via heme oxygenase-1 in human endothelial cells. Circ. Res..

[19] Beck D, Oda H, Shen S, Reinberg D (2012). PR-Set7 and H4K20me1: at the crossroads of genome integrity, cell cycle, chromosome condensation, and transcription. Genes Dev..

[20] Chen X (2018). SET8 is involved in the regulation of hyperglycemic memory in human umbilical endothelial cells. Acta Biochim. Biophys. Sin..

[21] Qi J (2020). High Glucose Induces Endothelial COX2 and iNOS Expression via Inhibition of Monomethyltransferase SETD8 Expression. J. Diabetes Res..

[22] Chen X (2020). High glucose inhibits vascular endothelial Keap1/Nrf2/ARE signal pathway via downregulation of monomethyltransferase SET8 expression. Acta Biochim. Biophys. Sin..

[23] Wang J (2020). High glucose mediates NLRP3 inflammasome activation via upregulation of ELF3 expression. Cell Death Dis..

[24] Shen X (2020). SET8 suppression mediates high glucose-induced vascular endothelial inflammation via the upregulation of PTEN. Exp. Mol. Med..

[25] Kumagai T (2014). Protein tyrosine phosphatase 1B inhibition protects against podocyte injury and proteinuria. Am. J. Pathol..

[26] Ito Y (2017). Protein tyrosine phosphatase 1B deficiency in podocytes mitigates hyperglycemia-induced renal injury. Metabolism.

[27] Schauer I (2010). CREB downregulation in vascular disease: a common response to cardiovascular risk. Arterioscler. Thromb. Vasc. Biol..

[28] Watson P (2010). Cardiac-specific overexpression of dominant-negative CREB leads to increased mortality and mitochondrial dysfunction in female mice. Am. J. Physiol. Heart Circ. Physiol..

[29] Tang X (2020). Resveratrol mitigates sevoflurane-induced neurotoxicity by the SIRT1-dependent regulation of BDNF expression in developing mice. Oxid. Med. Cell Longev..

