# Supplementary figures and images for: The CREB/KMT5A complex regulates PTP1B to modulate high glucose-induced endothelial inflammatory factor levels in diabetic nephropathy
Source: Cell Death Dis. 2021 Mar 29;12(4):333. doi: 10.1038/s41419-021-03629-4 (PMC8005662; doi:10.1038/s41419-021-03629-4)

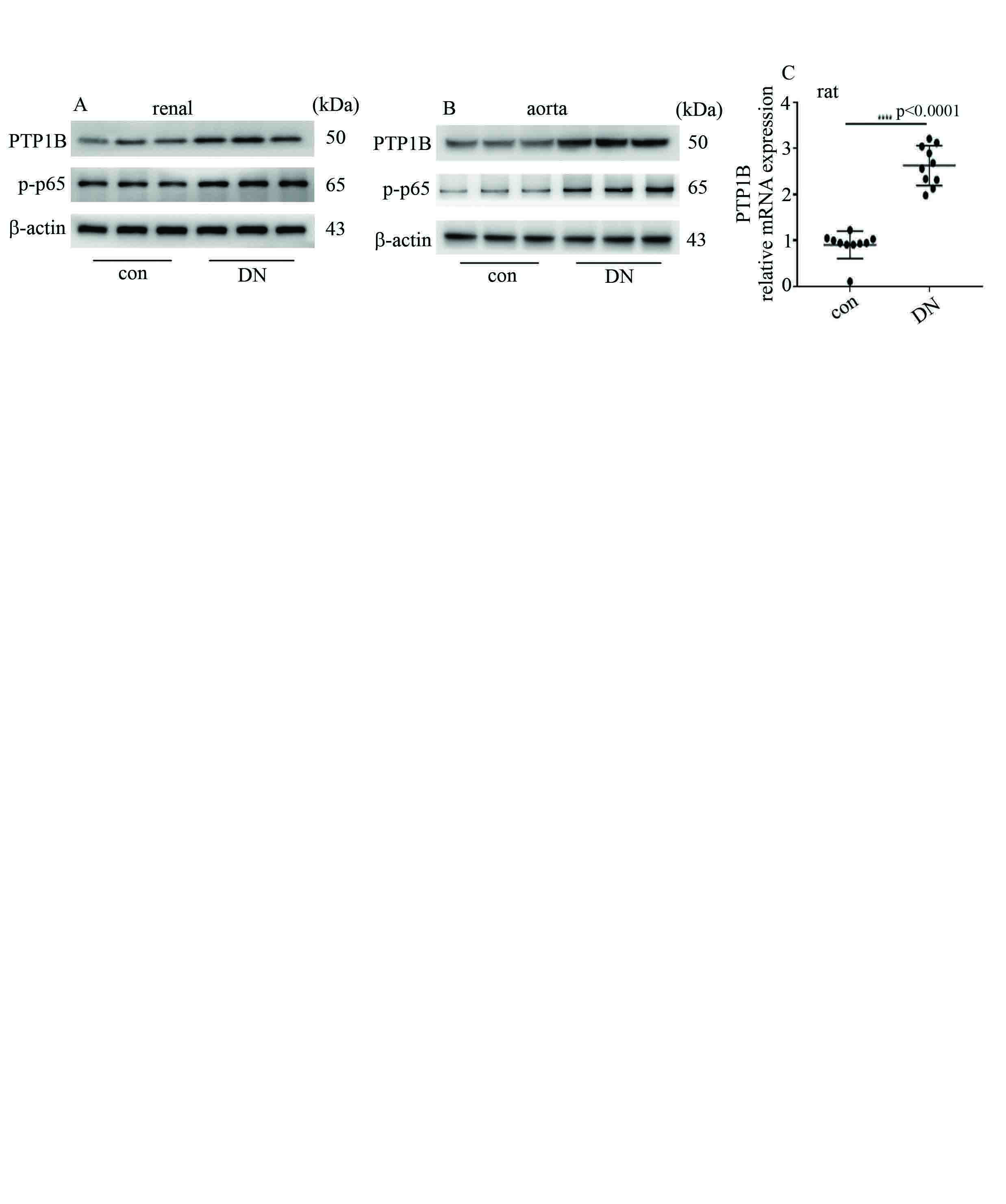

Supplement: Supplementary file 1 — Supplementary figure 1 [file 41419_2021_3629_MOESM1_ESM.tif]

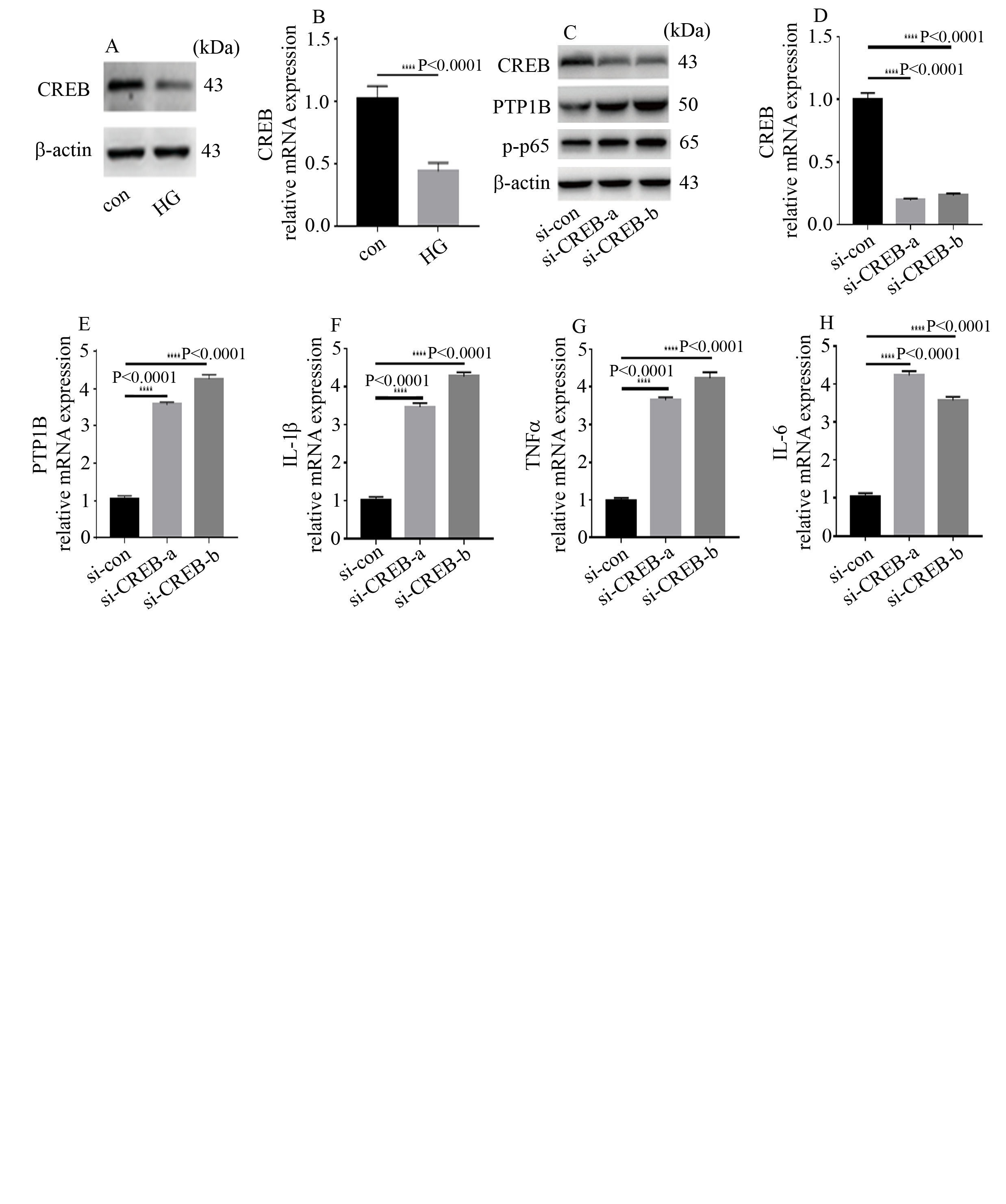

Supplement: Supplementary file 2 — Supplementary figure 2 [file 41419_2021_3629_MOESM2_ESM.tif]

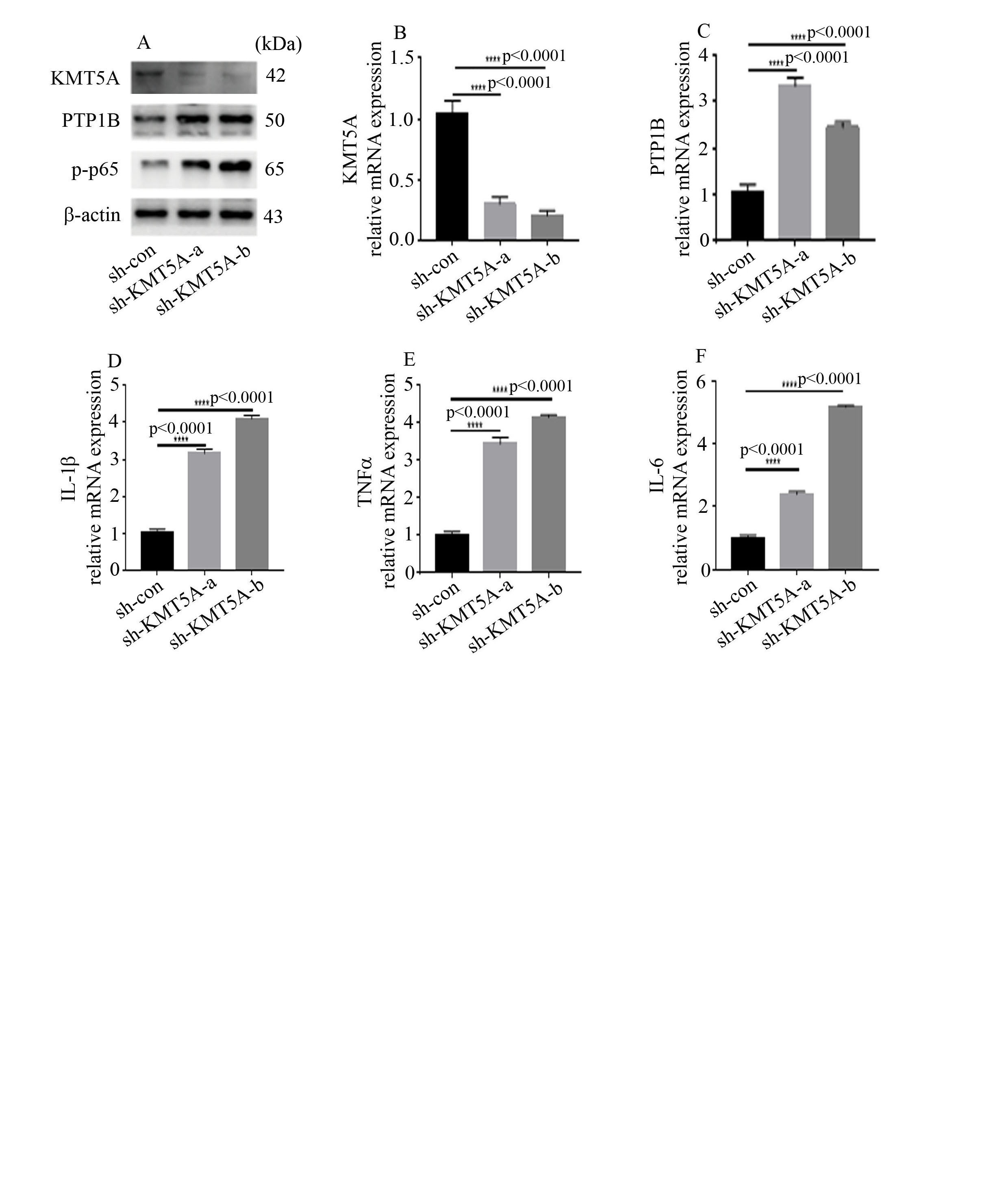

Supplement: Supplementary file 3 — Supplementary figure 3 [file 41419_2021_3629_MOESM3_ESM.tif]
