# Supplementary material for: The CREB/KMT5A complex regulates PTP1B to modulate high glucose-induced endothelial inflammatory factor levels in diabetic nephropathy
Source: Cell Death Dis. 2021 Mar 29;12(4):333. doi: 10.1038/s41419-021-03629-4 (PMC8005662; doi:10.1038/s41419-021-03629-4)
Supplement: Supplementary file 4 — supplementary figure legneds [file 41419_2021_3629_MOESM4_ESM.docx]

Supplementary Table 1. Primers used for real-time RT-PCR analysis.

Supplementary figure 1. PTP1B and p-p65 expression in renal and aorta of con and DN rats.

(A) Protein expression of PTP1B and p-p65 was tested by western blot in the renal tissues of the control rats and the DN rats. (B) Protein expression of PTP1B and p-p65 was tested by western blot in the aorta tissues of the control rats and the DN rats. (C) mRNA expression of PTP1B was tested by qPCR in the aorta tissues of the control rats and the DN rats. (* p<0.05, ** p<0.01, *** p<0.001, **** p<0.0001, n=10/group)

Supplementary figure 2. si-CREB elevated PTP1B expression, p65 phosphorylation and inflammatory factors levels in HUVECs.

(A) Results from the western blot analysis of CREB in the HUVECs with corresponding treatment. (B) mRNA expression of CREB was tested by qPCR with corresponding treatment. (C) Results from the western blot analysis of CREB, PTP1B and p-p65 in the HUVECs with corresponding treatment. (D-H) mRNA expression of CREB, PTP1B, IL-1β, TNFα and IL-6 were tested by qPCR with corresponding treatment. (* p<0.05, ** p<0.01, *** p<0.001, **** p<0.0001, n=5/group)

Supplementary figure 3. sh-KMT5A elevated PTP1B expression, p65 phosphorylation and inflammatory factors levels in HUVECs.

(A) Results from the western blot analysis of KMT5A, PTP1B and p-p65 in the HUVECs with corresponding treatment. (B-F) mRNA expression of KMT5A, PTP1B, IL-1β, TNFα and IL-6 were tested by qPCR with corresponding treatment. (* p<0.05, ** p<0.01, *** p<0.001, **** p<0.0001, n=5/group)
