# Supplementary material for: The CREB/KMT5A complex regulates PTP1B to modulate high glucose-induced endothelial inflammatory factor levels in diabetic nephropathy
Source: Cell Death Dis. 2021 Mar 29;12(4):333. doi: 10.1038/s41419-021-03629-4 (PMC8005662; doi:10.1038/s41419-021-03629-4)
Supplement: Supplementary file 5 — Supplementary Table 1 [file 41419_2021_3629_MOESM5_ESM.docx]

| species | RNA sequence |
| --- | --- |
| Human  β-actin  KMT5A  PTP1B  CREB  IL6  IL-1β  TNFα    Rat  β-actin  KMT5A  PTP1B  CREB | F 5’- CGGCTACAGCTTCACCACCAC -3’  R 5’- GCCATCTCTTGCTCGAAGTCCAG -3’  F 5’- TCCAGCAATCCTCCTCCTTCCTC -3’  R 5’- CCAGCCTAAGCAACAGATCCAGA -3’  F 5’- CCATATGGAGATGGAAAAGGAGTTCGAG-3’  R 5’- CCTAGTCCTCGTGGGAAAGCTCCTTCC -3’  F 5’- CCCAGCCATCAGTTATTCAG -3’  R 5’- GAGTTGGCACCGTTACAGTG-3’  F 5’- ACTCACCTCTTCAGAACGAATTG -3’  R 5’- CCATCTTTGGAAGGTTCAGGTTG -3’  F 5’- ATGAGCACTGAAAGCATGATC-3’  R 5’- TTAGGAAGACACAAATTGCATGGTGAACTCAGT-3’  F 5’- ATGAGCACTGAAAGCATGATC-3’  R 5’- TCACAGGGCAATGATCCCAAAGTAGACCTGCCC-3’  F 5’- CTTCCAGCCTTCCTTCCTGG -3’  R 5’- GAGCCACCAATCCACACAGA -3’  F 5’- GCAGGAAGAGAACTCCGTCG -3’  R 5’- AGAATCACATGACGGGGGTG -3’  F 5’- ACCCTGTGCGGAAATGCGGG-3’  R 5’- GCAGTCAGTCAACCCCGGC-3’  F 5’- TACAGGATAGACTAGCCACTT-3’  R 5’- AATATGTTTTCCTATCGGGGT-3’ |

Supplementary Table 1 Primers used for real-time RT-PCR analysis.
